# 3D-Printed Nacre-Inspired Polysaccharide Composite Films with Antibacterial Activity for Strawberry Preservation

**DOI:** 10.3390/foods15172956

**Published:** 2026-08-22

**Authors:** Shengsi Hu, Chenfeng Yu, Mei Xu, Leiqing Pan, Kang Tu

**Affiliations:** College of Food Science and Technology, Nanjing Agricultural University, Nanjing 210095, China; 2024108049@stu.njau.edu.cn (S.H.); 2023108046@stu.njau.edu.cn (C.Y.); 2025208004@stu.njau.edu.cn (M.X.); pan_leiqing@njau.edu.cn (L.P.)

**Keywords:** additive manufacturing, food packaging, zinc oxide nanoparticles, biomimetic design, fruit preservation

## Abstract

To overcome the limitations of conventional biopolymer films and reduce reliance on petroleum-based plastics, a nacre-inspired film was developed via 3D printing. During printing process, shear-induced alignment of mica flakes was achieved within a sodium alginate/xanthan gum matrix. Additionally, zinc oxide nanoparticles (ZnO NPs) were incorporated to achieve a synergistic reinforcement effect. Structural analysis revealed that the mica flakes within the film exhibited an oriented distribution, with ZnO NPs uniformly embedded in the interlayer voids, and hydrogen bonding assisted in forming a dense network of the components. Performance testing showed that the tensile strength rose from 13.8 MPa to 62.9 MPa. Improvements in water resistance and thermal stability were also observed. Furthermore, the material exhibited outstanding comprehensive protective properties, including a low water vapor permeability value of 7.587 × 10^−11^ g·m/m^2^·Pa·s, an ultraviolet blocking rate of 99.37% at a wavelength of 280 nm, and the ability to completely inhibit target bacterial strains, while also possessing good biodegradability and recyclability. Shelf-life tests indicated that the film fabricated in this work could notably prolong the shelf life of strawberries. Biocompatibility test results indicated that the film was safe and non-toxic, and showed no significant cytotoxicity.

## 1. Introduction

Freshly harvested vegetables and fruits lose moisture rapidly after harvest and are susceptible to microbial contamination, making them highly prone to spoilage and deterioration, which results in significant resource wastage and economic losses [1]. Food packaging films are used to preserve fresh produce by providing a barrier against external environmental factors. Yet conventional materials predominantly utilize petrochemical-based plastics, which do not undergo biological degradation, leading to plastic pollution and the accumulation of microplastics, posing an ongoing threat to the ecological environment [2,3]. Therefore, the development of renewable and biodegradable food packaging films has become an urgent research priority. Sodium alginate, a naturally derived linear anionic polysaccharide, combines biodegradability, safety, low cost, and excellent capacity for film formation [4], making it an optimal substrate for decomposable food packaging. Nevertheless, the mechanical properties of sodium alginate films alone are insufficient, and their sensitivity to moisture limits their application. Blending with polysaccharides such as xanthan gum can reinforce the polymer network and improve film stability [5]. However, composite films exhibit significant shortcomings in barrier properties, making it difficult to meet the demands of multifunctional food packaging.

The imitation of the pearl layer structure provides an effective design strategy for improving the performance of polysaccharide-based composite films [6]. The natural pearl layer is composed of micrometer-sized calcite plates alternatingly stacked with an organic matrix, forming a typical “brick-and-mortar” layered structure. This structure significantly enhances the barrier performance and mechanical strength of the film by extending the molecular diffusion path and promoting stress dispersion [7,8]. In the process of biomimetic construction, the two-dimensional sheet components play a crucial role as “brick” units. In recent years, inorganic nanosheets such as boron nitride, graphene, MXene, molybdenum disulfide, layered double hydroxides, and montmorillonite [9,10,11,12] have been used to construct the imitation pearl layer. In contrast, mica, as a natural layered silicate mineral, possesses excellent thermal stability, mechanical properties, cost advantages, and ultraviolet shielding ability, and shows good application potential in biomimetic structure construction. However, since both polysaccharides and mica do not have inherent antibacterial activity, the application potential of the composite film is still limited.

Zinc oxide nanoparticles (ZnO NPs) are efficient and broad-spectrum inorganic antibacterial agents, featuring excellent thermal stability and good biological safety. The US Food and Drug Administration has classified them as a generally considered safe substance [13] and approved for application in food contact materials. Their antibacterial mechanism mainly includes the generation of reactive oxygen species (ROS), the release of Zn^2+^, and the direct contact of the nanoparticles with the cell membrane [14]. Additionally, as zero-dimensional nanomaterials, ZnO NPs can be uniformly dispersed in polysaccharide matrices, effectively filling the tiny pores of two-dimensional mica sheets and polymers [15], which can further enhance the density and barrier function of the film structure.

When fabricating nacre-like films, controlling the orientation of two-dimensional (2D) materials is key to enhancing the mechanical and gas barrier properties of films. Currently, the orientation control of 2D nanofillers in films primarily relies on strategies such as self-assembly [16], spin coating [17], vacuum filtration [18], magnetic field induction [19], and mechanical post-stretching [20]. However, these methods generally suffer from limited orientation control, low fabrication efficiency, and a tendency to introduce microdefects such as pores and agglomeration. Furthermore, some methods require additional modification or post-processing steps. For instance, magnetic field induction necessitates magnetic modification, while mechanical post-stretching can even lead to deformation or rupture of the film, thereby limiting the stable construction of nacre structures. In contrast, 3D printing allows shear forces during the extrusion process to directly induce the ordered alignment of 2D nanosheets along the printing direction [21] without the need for additional modification or post-processing. Furthermore, compared to traditional cast film methods, 3D printing can significantly reduce drying time due to the high solid content of its ink, while enabling one-step fabrication of multilayer structures [22]. At the same time, its controllable structural design demonstrates broad application potential in film preparation [23]. Nevertheless, there remains insufficient systematic knowledge on how to enable food packaging films to simultaneously form the biomimetic nacre-like structure and possess antimicrobial properties during the 3D printing process. In light of this, this work strives to overcome the performance limitations of pure SW films via 3D-printed nacre-mimetic structures with ZnO NPs, providing both an effective preservation strategy and a biodegradable substitute for conventional films.

## 2. Materials and Methods

### 2.1. Materials

Sodium alginate (80 mesh, viscosity 570–800 mPa·s) was acquired from Shandong Youso Chemical Technology Co., Ltd. (Linyi, China). Mica (99.5% purity, 200 mesh) was purchased from Guzhang County Shanlin Stone Language Mineral Products Co., Ltd. (Guzhang, China). Xanthan gum (80 mesh), ZnO NPs (particle size < 100 nm), and glycerin were purchased from Shanghai Macklin Biochemical Technology Co., Ltd. (Shanghai, China). The fresh strawberries were purchased at a local market in Nanjing.

### 2.2. Preparation of Composite Inks

In a beaker containing 90 mL of pure water, 2.5 g of sodium alginate (SW), 1.5 g of xanthan gum (XG) and 2.4 g of glycerol were added, and the mixture was stirred continuously at 65 °C until completely dissolved. Meanwhile, 2 wt% ZnO NPs (based on the mass of the polysaccharide) and 1 g of mica were weighed and dispersed in 10 mL of ethanol. After 30 min of treatment with 200 W ultrasound, a homogeneous dispersion was obtained, which was added to the polysaccharide mixture and stirred until uniform. The resulting inks were centrifuged at 3200 rpm to remove air bubbles, then transferred to print cartridges for subsequent 3D printing. For ease of discussion, the films were named according to the order of component addition: SW represents the sodium alginate matrix, while SX, SXM, and SXMZ films represent the composite films obtained by sequentially adding xanthan gum, mica, and ZnO NPs, respectively.

### 2.3. Preparation of Composite Films

The experiment utilized an extrusion-type 3D printer (FOODBOT-DM1, Hangzhou Shiyin Technology Co., Ltd., Hangzhou, China) equipped with a 0.8 mm diameter nozzle. The print parameters were set to 100% infill density, a 45° infill angle, and two layers. The infill pattern for the first layer was concentric circles, and for the second layer, straight lines. The print speed was set to 27 mm/s. For 3D printing model design, the dimensions of the films for performance testing and food storage were set at 60 mm × 60 mm × 0.16 mm and 120 mm × 120 mm × 0.16 mm, respectively. After printing, the wet films were placed in an oven set to 45 °C and left to dry for 3 h. After drying, they were taken out and temporarily stored at 25 °C and 50% relative humidity (RH) for subsequent testing.

### 2.4. Structural and Property Characterization

#### 2.4.1. Rheological and Print Accuracy Characterization of Inks

The rheological properties of the printing inks were examined using a rotational rheometer (Anton Paar MCR 302e, Graz, Austria). The measurements were performed using a parallel-plate rotor with a diameter of 50 mm and a gap width of 1 mm. The viscosity data were collected over a shear rate range of 0.1–100 s^−1^, and a viscoelastic characterization scan was performed over an angular velocity range of 1–100 rad/s, recording the changes in the storage modulus (G′) and loss modulus (G″) as a function of frequency. Printing accuracy was evaluated by comparing the areas of the model and the fabricated film specimens, following an approach described previously [24].

#### 2.4.2. Thickness Measurement

Thickness (mm) was measured at least five times using an electronic micrometer (Greenery, Yantai, China).

#### 2.4.3. Scanning Electron Microscopy (SEM) Test

The microstructure of all the films was characterized with a field-emission SEM (Regulus, Hitachi, Japan). To further elucidate the structure of the SXMZ film, energy-dispersive X-ray spectroscopy (EDS) was performed to examine the elemental distribution within the cross-sections.

#### 2.4.4. Fourier Transform Infrared (FTIR) Spectroscopy Test

FTIR spectra of the films were obtained using an attenuated total reflection (ATR) FTIR spectrometer (Nicolet iS20, Thermo Fisher Scientific, Waltham, MA, USA). The wavenumber range was set to 4000–400 cm^−1^, with a resolution of 4 cm^−1^ and 32 scans.

#### 2.4.5. X-Ray Diffraction (XRD) Analysis

The crystal structure was analyzed using an X-ray diffractometer (SmartLab SE, Rigaku, Tokyo, Japan) with a 2θ range of 5–90° and a scanning rate of 10°/min.

#### 2.4.6. Determination of Water Resistance

Water content (WC) and water solubility (WS) were determined as described in previous reports [25]. A contact angle measuring machine (Dataphysics OCA 20, Filderstadt, Germany) was employed to evaluate the water contact angle (WCA). The measurement used 4 μL of water stabilized for 10 s.

#### 2.4.7. Optical Performance Test

A TU-1900 UV-Vis spectrophotometer (Persee, Beijing, China) was adopted to analyze the optical properties of the samples.

#### 2.4.8. Water Vapor Permeability (WVP) Test

WVP values were tested using the procedure outlined by a published work [26]. Specifically, 30 mL of pure water was placed in a plastic tube, sealed with a test film, and left to stand for 24 h at 25 °C and 50% RH. Equation (1) was employed to compute the WVP value.

(1) $$WVP\left(g\cdot m/m^{2}\cdot Pa\cdot s\right)=\frac{∆m\times d}{∆t\times A\times ∆P}$$

In the equation, ∆m/∆t (g/s) represents the mass variation per unit time, d (m) represents the film thickness, A (m^2^) represents the effective area of the film, and ∆P (Pa) represents the vapor pressure drop across the film under the test conditions.

#### 2.4.9. Mechanical Property Characterization

Tensile testing was performed on rectangular strips (10 × 60 mm) using a TA-XT plus texture analyzer (Stable Micro Systems, Godalming, UK) with an articulated tensile grip, at a test speed of 0.8 mm/s [27]. Equations (2) and (3) were used to determine the tensile strength (TS) and elongation at break (EAB), respectively.

(2) $$TS\left(MPa\right)=\frac{F}{d\times w}$$

In the equation, F (N) represents the maximum tensile load exerted on the film when it breaks, d (mm) represents the film thickness, and w (mm) represents the film width.

(3) $$EAB\left(\%\right)=\frac{L-L_{0}}{L_{0}}\times 100$$

In the equation, L (mm) represents the maximum elongation of the film at break, and L_0_ (mm) denotes the initial length of the film.

#### 2.4.10. Thermal Stability Analysis

A thermal gravimetric analyzer (Netzsch STA 449f5, Selb, Germany) was used to investigate the thermal stability of the samples. The samples were heated from 30 °C to 800 °C at a rate of 10 °C/min in an N_2_ atmosphere, and the mass loss curves were recorded.

#### 2.4.11. Antibacterial Activity Assessment

The antimicrobial function of the films was evaluated using the method from established protocols [28]. Specifically, 2 cm × 2 cm samples were sterilized and then incubated with suspensions of *Escherichia coli* and *Staphylococcus aureus* (10^5^–10^6^ CFU/mL) under shaking conditions at 37 °C for 24 h. After incubation, 100 μL of the suspension was uniformly distributed onto nutrient agar plates and cultured at 37 °C for 16 h.

#### 2.4.12. Safety Assessment

For cell compatibility testing, we referred to the prior study [29]. L-929 cells were inoculated into a 96-well plate at a density of 1 × 10^5^ cells/mL, with 100 μL per well. After the cells had adhered, they were co-cultured with the pre-sterilized films (10 mg) for 24 h or 48 h. Afterwards, the absorbance was measured at 450 nm using the cell counting kit 8 (CCK-8) assay.

#### 2.4.13. Recyclability Test

A total of 4 g of the waste SXMZ film generated during the experiment was collected, shredded, and introduced into 100 mL of pure water and 5 drops of glycerin. The mixture was stirred continuously at 60 °C to form a casting solution. 13 g of the solution was transferred into a plastic Petri dish with a diameter of 10 cm, and the regenerated films were produced after drying at 55 °C for 6 h. The key performance parameters of the regenerated films were then evaluated.

#### 2.4.14. Biodegradability Assessment

Film samples were buried in soil at the Binjiang Campus of Nanjing Agricultural University at a depth of 10 cm. During the test, water was periodically added to maintain adequate soil moisture. Samples were retrieved at predetermined intervals to observe morphological changes and were photographed for documentation, and the degradation behavior was analyzed based on the macroscopic variations.

#### 2.4.15. Preservation of Strawberries

Fresh strawberries were placed in plastic containers and sealed with composite films, while unpackaged fruit acted as the control group (CK). The storage conditions were set to 25 °C and 50% RH. Fruits were taken periodically to determine their weight loss rate using Equation (4). Meanwhile, firmness was determined on a texture apparatus (TMS-pro, FTC, Sterling, VA, USA) using a 5 mm cylindrical probe at the equatorial region of strawberries. Testing was conducted at a trigger force of 0.4 N, a test speed of 60 mm/s, a penetration depth of 5 mm, and a return speed of 300 mm/s.

(4) $$Weight\;loss\left(\%\right)=\frac{m_{1}-m_{2}}{m_{1}}\times 100$$

where m_1_ (g) represents the initial mass, and m_2_ (g) refers to the mass at a given storage time.

### 2.5. Data Analysis

Each experiment was repeated at least three times. Data are presented as mean ± standard deviation. Comparisons between groups were conducted using one-way analysis of variance (ANOVA) together with Duncan’s post hoc test using SPSS 22.0 (IBM Corp., Armonk, NY, USA). Figures were created using Origin 2024 (Origin Lab Co., Northampton, MA, USA). The level of statistical significance was set at *p* < 0.05.

## 3. Results and Discussion

### 3.1. Rheology and Printability Analysis

**Figure 1:**
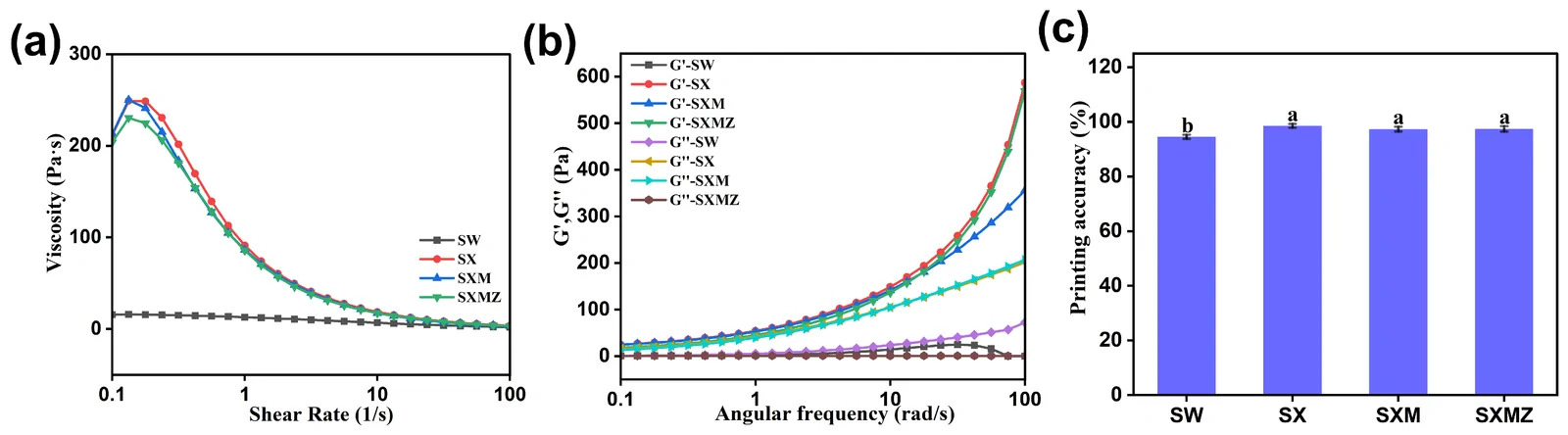
(**a**) Apparent viscosity, (**b**) G′, and G″ of printing inks. (**c**) Print accuracy. Different letters (a,b) indicate significant differences between groups (*p* < 0.05).

Figure 1a presents the viscosity versus shear rate curves for inks with different compositions. All formulations exhibited typical shear-thinning behavior, whereby viscosity gradually decreased as the shear rate increased. This facilitated the smooth extrusion of the inks through the needle and contributed to preventing nozzle clogging. Furthermore, the addition of XG significantly enhanced the system’s viscosity, which may be attributed to the formation of hydrogen bonds that strengthened the gel network [30]. However, the addition of mica caused a slight decline in the ink’s viscosity in the low-shear region. This was probably due to mica disrupting the interactions between the two polysaccharide molecules [31], whereas ZnO NPs enhanced the viscosity in this region by forming hydrogen bonds with other components. Figure 1b showed the variation in G′ and G″ of each ink formulation as a function of angular frequency. For the SW ink, G″ consistently remained higher than G′ across the entire frequency range, behaving as a viscous fluid. In contrast, for the remaining samples, G′ consistently remained higher than G″, demonstrating a predominantly elastic, solid-gel-like behavior. This shift indicated that the addition of other components enhanced the inks’ cohesion, facilitating shape retention. In line with the rheological analysis, Figure 1c demonstrated that the printing accuracy of the SX, SXM, and SXMZ inks was significantly superior to that of the pure SW group, further validating the improvement in printability achieved through component optimization. The low standard deviation also reflected good stability and repeatability of this 3D film-fabrication process.

### 3.2. Microtopography Analysis

**Figure 2:**
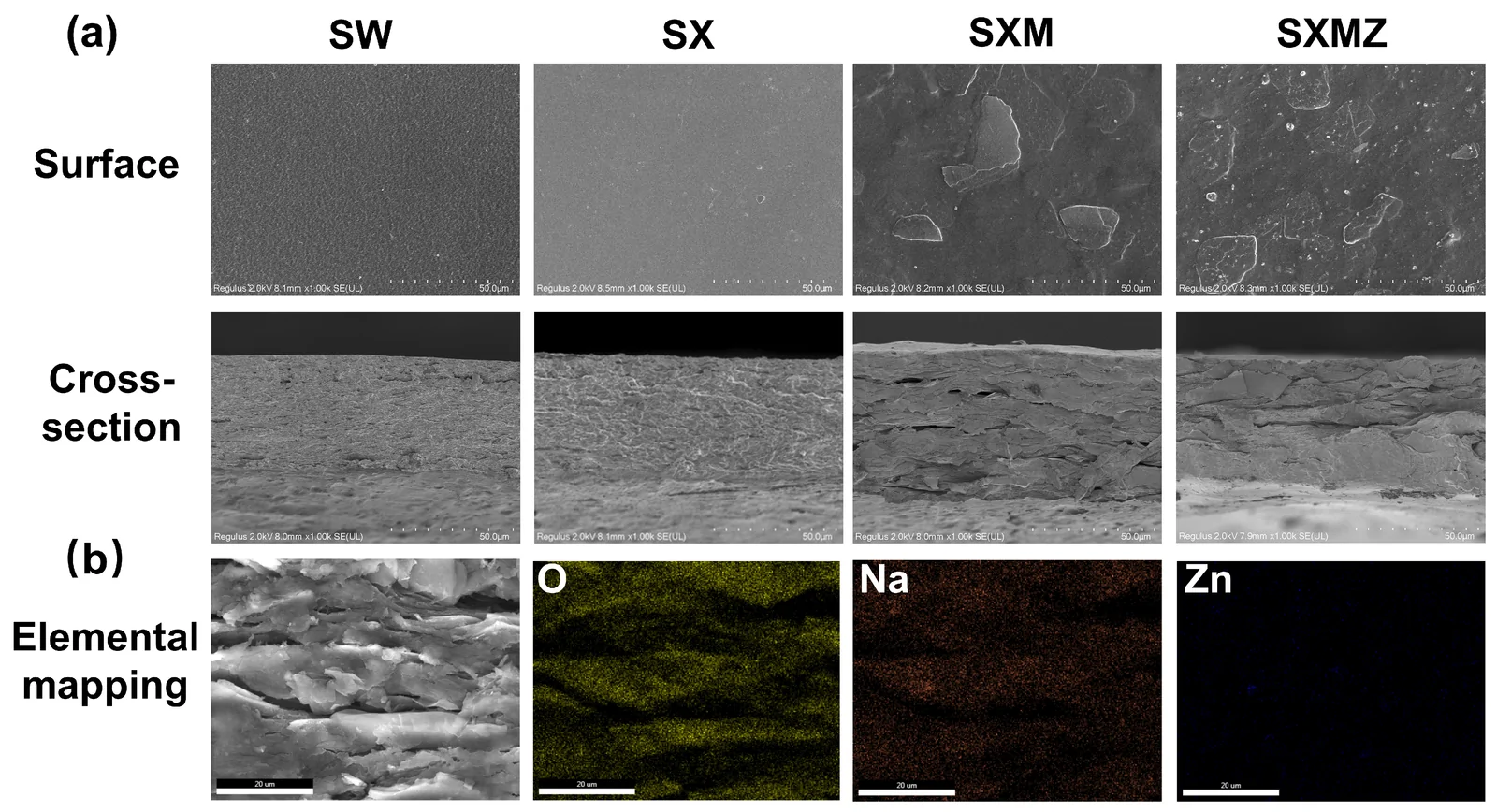
(**a**) SEM images of all film samples. (**b**) Cross-sectional photograph of the SXMZ film and the distribution of oxygen, sodium, and zinc in the corresponding regions.

The microscopic photographs of the prepared films are shown in Figure 2a. The results show that the SW and SX films had continuous and dense surface structures, indicating their excellent film-forming performance. In contrast, after the addition of mica and ZnO NPs, flake-like and dot-like filler structures were visible on the outer surface of the films.

The cross-sections of SW and SX films exhibited dense and uniform structures. However, the voids observed in the cross-section of the SXM film stemmed from mica that remained unexfoliated, while the introduction of ZnO NPs effectively filled these defects and densified the film structure. Exfoliation of mica could improve its dispersion in the matrix and reduce structural defects. Nevertheless, previously reported exfoliation methods typically involved high energy consumption, low yield, and a tendency to compromise the integrity of the flake structure and reduce the aspect ratio [32,33]. The unexfoliated mica flakes maintained a relatively high aspect ratio, sufficient to act as effective fillers for reinforcement and barrier functions. In addition, the shear flow field during 3D printing was confirmed to effectively regulate the orientation of such high-aspect-ratio fillers. For example, in a study on 3D-printed sodium alginate/graphene oxide composites, the Hermans orientation factor (f) of the ink initially approached 0, and after nozzle shear, extrusion, and drying, f increased to 0.78 [34], which fully demonstrated the effectiveness of 3D printing in regulating the orientation of two-dimensional fillers. These results were further supported by elemental mapping analysis of the SXMZ film cross-section (Figure 2b), which showed that the characteristic elements O and Na exhibited a layered distribution, while Zn had a lower concentration and was uniformly distributed across the cross-section. In summary, this study successfully constructed nacre-like hierarchical structures in the composite film, with flake mica oriented as a rigid “brick” layer, a polysaccharide cross-linked network filling the gaps as a ductile “mortar” phase, and ZnO NPs further increasing interfacial density.

### 3.3. ATR-FTIR Results

**Figure 3:**
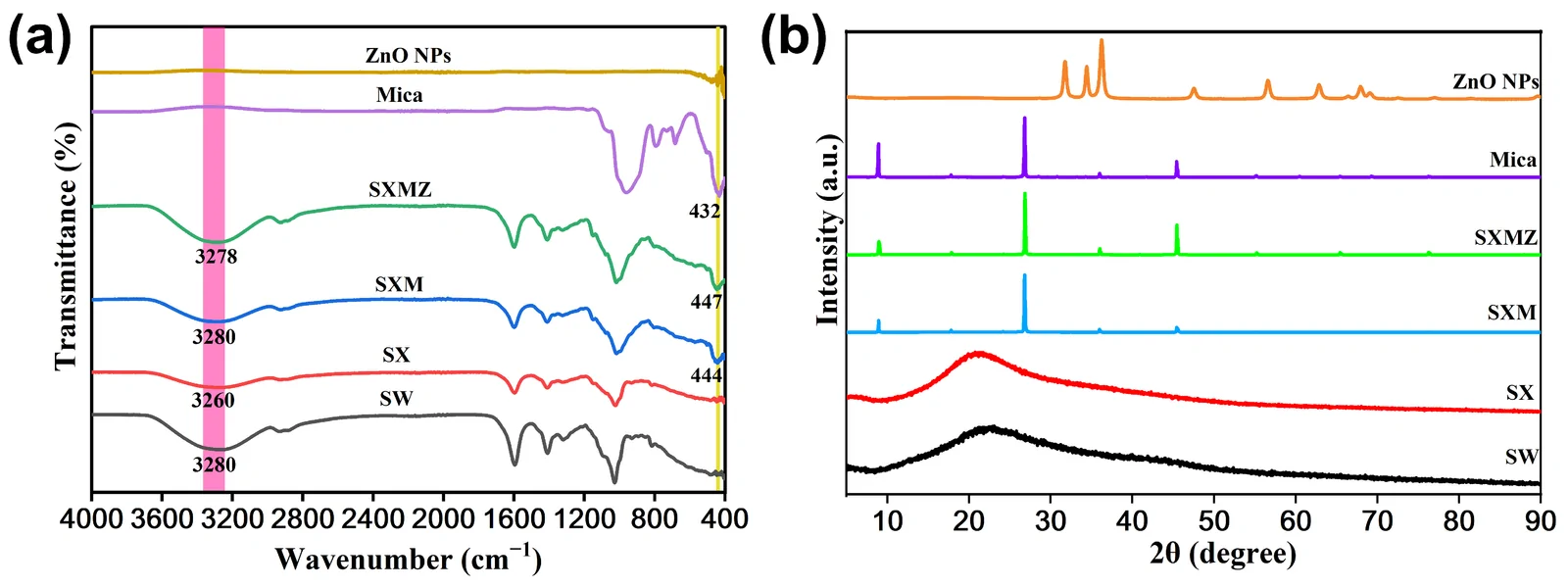
(**a**) FTIR spectra and (**b**) XRD patterns of mica, ZnO NPs, and the prepared film samples.

ATR-FTIR spectroscopy was used to detect the functional groups in the composite films and the interactions between their components. As shown in Figure 3a, the SW film spectrum exhibited characteristic peaks of polysaccharides. The O–H stretching vibration peak was located at 3280 cm^−1^, the C–H stretching peak was at 2929 cm^−1^, the asymmetric –COO− stretching peak was at 1594 cm^−1^, the symmetric –COO− stretching peak was at 1407 cm^−1^, and the C–O–C glycosidic bond stretching peak was at 1025 cm^−1^ [35]. Upon introduction of XG, mica, and ZnO NPs, the –OH absorption peak shifted and underwent intensity changes, which were attributed to the construction of hydrogen bonds among the components [36,37,38]. Additionally, the SXM and SXMZ composite films exhibited typical absorption bands at around 445 cm^−1^, assignable to the Si–O bending vibration of mica [39,40], confirming that the mica was successfully embedded within the polymeric framework. The remaining characteristic peaks remained essentially unchanged, and no new chemical bonds were formed, indicating that no significant chemical reaction occurred in the system, which was primarily constructed as an overall network through non-covalent interactions.

### 3.4. XRD Analysis

The variations in the crystal structure of the thin films were examined using X-ray diffraction. As shown in Figure 3b, the SW film displayed a wide diffraction peak near 2θ = 22°, indicating that the SW matrix was predominantly in a typical amorphous state [41]. After mixing with XG, this broad peak shifted slightly toward smaller angles, indicating that the interaction between the two polysaccharide molecules altered the molecular arrangement within the SW matrix and restructured the polymer network [42]. In addition, when mica flakes were incorporated into the system, the composite films exhibited distinct diffraction peaks at 2θ = 8.9°, 26.8°, and 45.4°, assigned to the (003), (009), and (0015) crystal planes [43] of mica, respectively. The incorporation of mica into the films created crystalline regions that were difficult to penetrate, which was anticipated to enhance the films’ barrier performance. Furthermore, following the incorporation of ZnO NPs, no additional diffraction peaks were detected in the diffraction spectrum of the film sample. This was because the ZnO NPs were uniformly distributed, so no detectable crystalline phases had formed [44].

### 3.5. Analysis of Water Resistance

**Figure 4:**
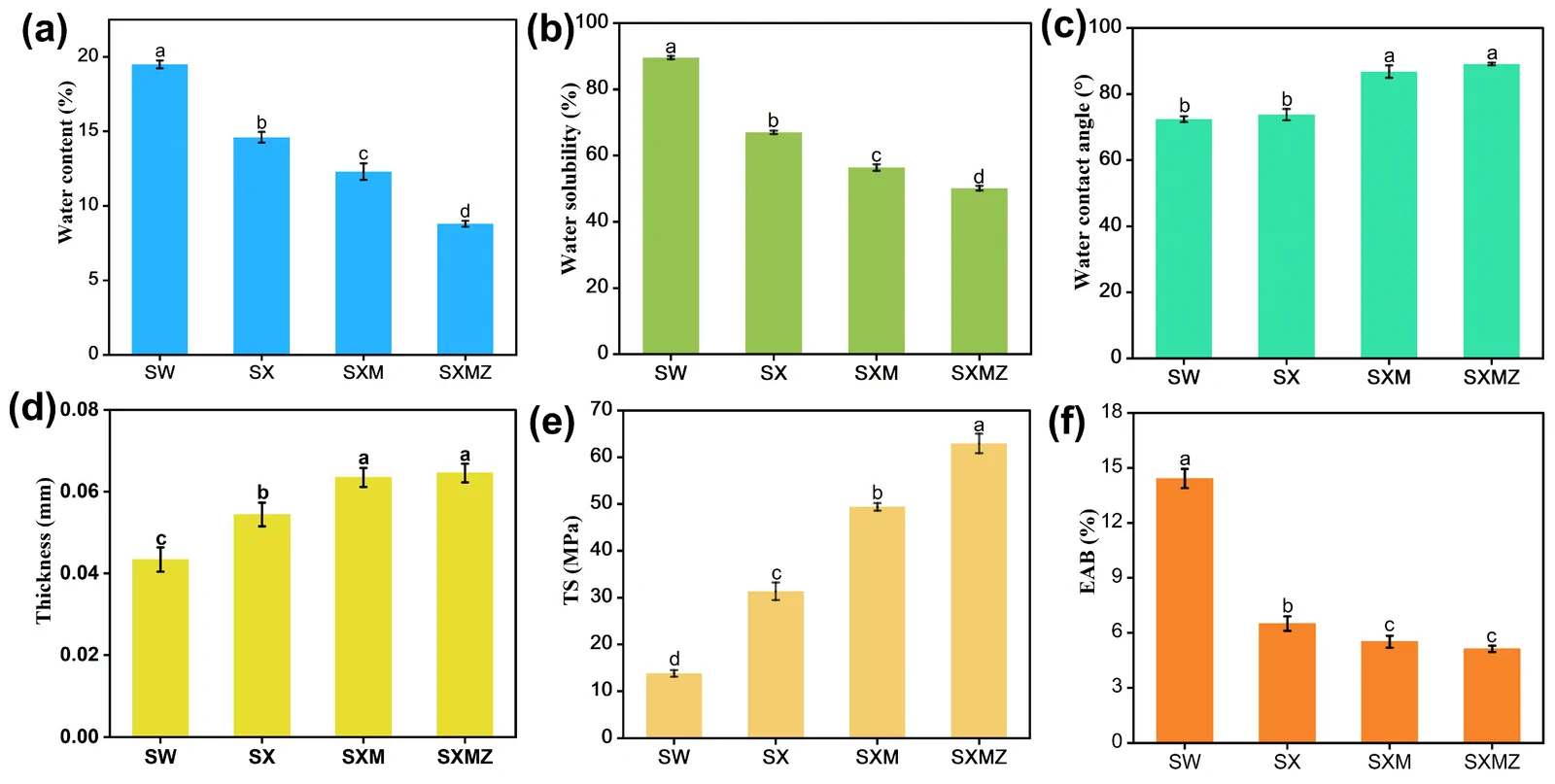
(**a**) WC, (**b**) WS, and (**c**) WCA of the prepared films. (**d**) Thickness, (**e**) TS, and (**f**) EAB of the films. Different letters (a–d) indicate significant differences between groups (*p* < 0.05).

In high-humidity environments, the structural stability of biobased films is largely determined by their water resistance. As shown in Figure 4a–c, with the gradual introduction of components, the WC of the composite films decreased significantly, and WS dropped sharply from 89.6% in the SW film to 50.2% in the SXMZ film. Such performance improvements were primarily ascribed to the reinforcing effect of inorganic constituents on the fine structure of the polysaccharide matrix. Mica flakes and ZnO NPs in the matrix, on the one hand, reduced the system’s free volume through filling [45], thereby limiting the retention and permeation of water molecules. On the other hand, they enhanced interfacial interactions between components, which encouraged certain hydrophilic groups (such as –OH) to participate in the construction of a more compact network structure, thereby reducing the accessibility of water molecules and suppressing the swelling and dissolution behavior of the films. Furthermore, the WCA values increased from 72.4° to 89.1°, indicating that the film surfaces became more hydrophobic. This was primarily attributed to the rough surface structure created by inorganic fillers on the film surface, as well as their relatively low surface energy, which reduced the spreading ability of water droplets on the film’s surface. In summary, the introduction of mica and ZnO NPs to construct a nacre-like structure effectively enhanced the water resistance, providing a material foundation for their practical application under high-humidity or cold-chain conditions.

### 3.6. Thickness and Mechanical Performance

Figure 4d demonstrated that a stepwise elevation in the thickness of the composite films was observed as components were introduced. The increase was attributed, on the one hand, to the increment in the solids level within the mixture [31], and on the other hand, to the formation of a nacre-like layer framework that supported the longitudinal structure of the films during the drying process, thereby restricting vertical shrinkage.

Subsequently, a detailed investigation of the mechanical properties of the composite films was conducted. As illustrated in Figure 4e,f, with the formation of the composite system, the mechanical properties of the films underwent notable changes. Specifically, the TS continued to increase, while the EAB gradually decreased. The SXMZ film manifested the most outstanding mechanical performance, with a TS of 62.9 MPa, far exceeding the 13.8 MPa of the pure SW film. The enhancement in mechanical properties originated from the synergistic effects of strong hydrogen bonding between components and the brick-and-mortar structure. Highly oriented mica layers effectively dispersed the load by inducing crack deflection [46]. Furthermore, the introduction of ZnO NPs filled microscopic voids and strengthened interfacial bonding and structural density by creating additional cross-linking sites [47], thereby enabling more continuous stress transfer. However, a decline in the EAB occurred alongside this. This phenomenon arose from the network of hydrogen bonds combined with the presence of inorganic fillers, which restricted the slippage of molecular chains.

### 3.7. Thermal Properties

**Figure 5:**
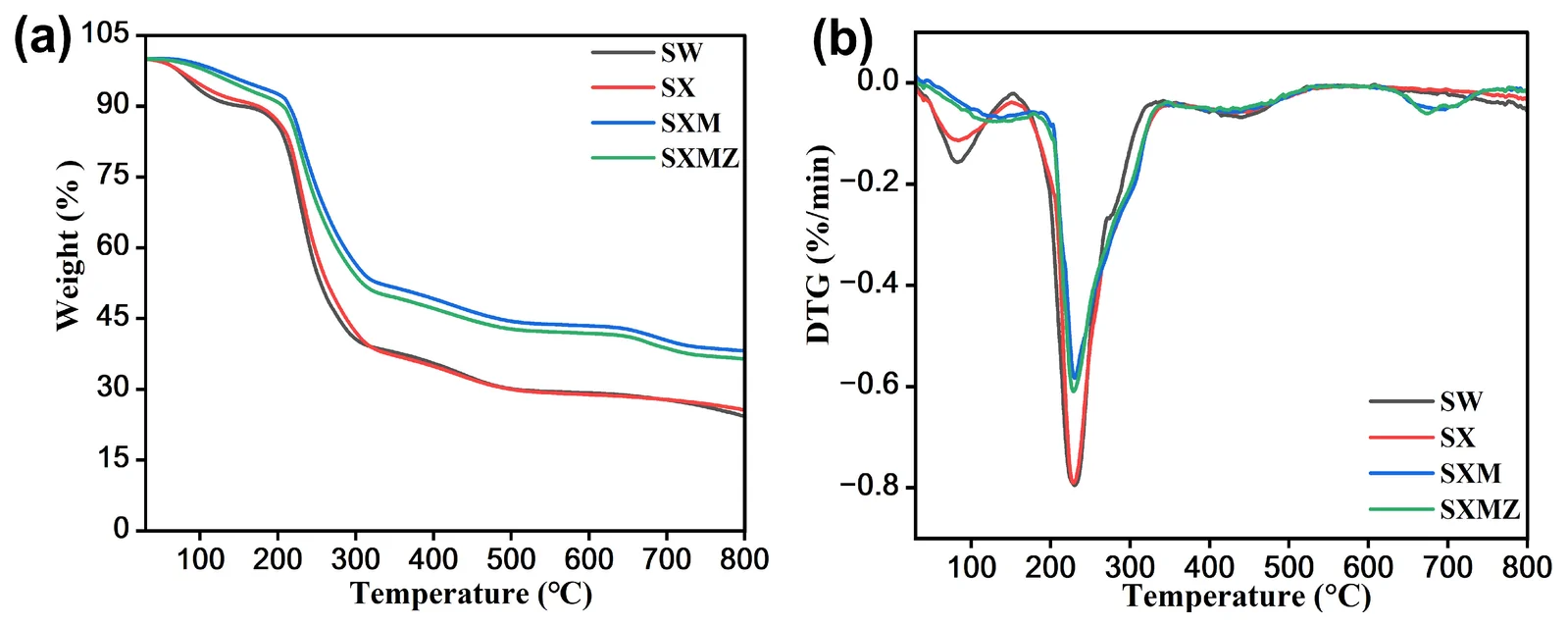
(**a**) Thermogravimetric curves and (**b**) derivative thermogravimetric (DTG) curves of films.

As depicted in Figure 5, the thermal degradation behavior of samples could be separated into three phases. The initial step (below 150 °C) was linked to the elimination of physically adsorbed and bound water from the system. During the subsequent main decomposition phase (150–350 °C), the plasticizer glycerol underwent thermal volatilization, while heat-sensitive groups in the polysaccharide matrix underwent dehydration, condensation, and preliminary decomposition. The final stage (above 350 °C) corresponded to the deep transformation of the heat-resistant organic residual framework into carbonized products. Comparative test results demonstrated that the thermal residue of film samples was significantly improved after the incorporation of mica flakes. Specifically, the residual content of the pure SW film at 800 °C was only 24.2%, whereas that of the SXMZ composite film increased to 36.4%. This was attributed to the formation of hydrogen bonds and the thermal residue of heat-resistant inorganic fillers. It was worth noting that the ZnO NPs catalyzed the decomposition of organic components to some extent [28]. Nevertheless, the thermal stability of the SXMZ group still outperformed that of the systems without an inorganic phase, as the physical support effect of layered mica dominated. Findings revealed that the formation of a nacre-like structure contributed to higher thermal resistance.

### 3.8. Barrier Properties

#### 3.8.1. UV Barrier Properties

**Figure 6:**
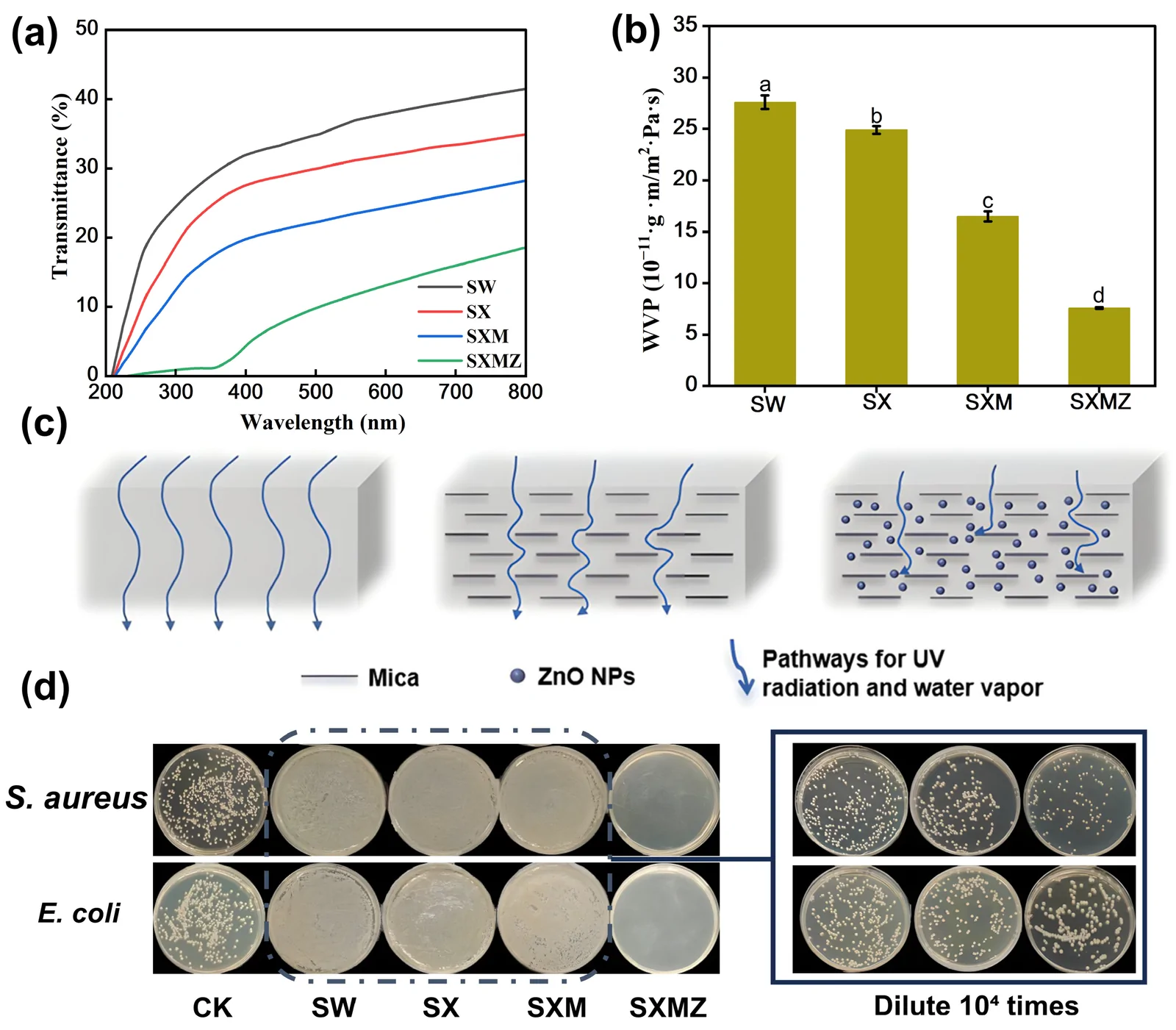
(**a**) Optical performance and (**b**) WVP of samples. (**c**) Schematic of the barrier enhancement mechanism. (**d**) Antibacterial effect against *S. aureus* and *E. coli* of the film samples. Different letters indicate significant differences between groups (*p* < 0.05).

During the preservation of fresh produce, photooxidation caused by ultraviolet (UV) radiation accelerates the breakdown of nutrients and tissue aging. Therefore, the UV barrier performance of films is a key indicator for examining their capability to preserve freshness. Figure 6a illustrates the optical characteristics of composite films. As a layered structure resembling a nacreous layer gradually formed, the UV shielding efficacy of the resulting films was considerably elevated. In particular, the SXMZ film achieved a blocking percentage of 99.37% at a wavelength of 280 nm. This was mainly attributed to intense physical reflection and scattering from regularly arranged mica interfaces as well as the UV absorption of wide-bandgap ZnO NPs, whose synergistic effects achieved nearly full UV shielding.

#### 3.8.2. WVP

A critical factor leading to postharvest food quality deterioration is moisture loss, as it can exacerbate tissue dehydration and textural degradation. Therefore, reducing WVP is crucial for suppressing moisture migration and delaying the deterioration process. However, pure polysaccharide films have limited moisture-barrier properties because their molecular chains are rich in hydrophilic groups, which easily form continuous channels for water diffusion. As displayed in Figure 6b, with the introduction of mica and ZnO NPs, a steady decline in WVP was observed, with the SXMZ film achieving the lowest value (7.587 × 10^−11^ g·m/m^2^·Pa·s). This performance enhancement was primarily attributed to the maze effect created by the mica flakes, which lengthened the diffusion path of water molecules [48]. Although ZnO NPs are inherently hydrophilic, their dense crystalline regions exhibited low permeability to water molecules and further hindered the permeation of water [49]. At the same time, the filling effect of the inorganic fillers and the hydrogen bonds formed between them and the polysaccharide chains further narrowed the permeation channels for water molecules, thereby significantly reducing the WVP. Figure 6c illustrated how these two fillers worked together to strengthen the barrier features of the composite films.

### 3.9. Antimicrobial Properties

The results of the antibacterial tests on *S. aureus* and *E. coli* using the film samples were given in Figure 6d. Compared with the blank control (CK), the test films without ZnO NPs demonstrated an increase in colony count of approximately 4 orders of magnitude, indicating a lack of antimicrobial activity and even promotion of bacterial proliferation. This stemmed from the partial hydrolysis of the polysaccharide matrix during incubation, which released available carbon sources and thereby promoted bacterial growth and metabolism. In contrast, the SXMZ composite film exhibited complete inhibition of the target bacteria. This enhanced performance stemmed from the direct contact of ZnO NPs with bacterial cells, as well as the release of Zn^2+^ and ROS. Direct contact disrupted membrane permeability, leading to the efflux of intracellular substances. In addition, Zn^2+^ penetrated bacterial cells and bound to the sulfhydryl groups of proteins, disrupting bacterial metabolism by inhibiting gene replication and enzyme activity [50]. Meanwhile, the resulting ROS caused oxidative damage to bacterial structures [51]. In addition to the two strains selected above, ZnO NPs or their composites also exhibited strong inhibitory effects against strains such as *Listeria innocua* [52], *Bacillus cereus* [53], *Aspergillus flavus* [54], and *Botrytis cinerea* [55]. This highly effective antimicrobial activity overcame the susceptibility of bio-based packaging materials to microbial contamination, confirming the key contribution of the incorporation of ZnO NPs.

### 3.10. Performance Analysis and Comparison

Given the partial overlap in material composition and film-forming processes, this section compared the SXMZ film with those developed in recent years that used SW, XG, mica, or ZnO NPs as raw materials or employed 3D printing as the film-forming method. In addition, to comprehensively evaluate the enhancing effect of the nacre-like structure reinforced by ZnO NPs on the properties of the pure SW film, we also compared it with the unmodified films. In terms of barrier performance, the UV blocking efficiency of the pure SW film at 280 nm was only 77.77%. After modification with a simulated nacre structure, the SXMZ film’s efficiency rose to 99.37%, which was comparable to the levels reported in most of the literature listed in Table 1. Water vapor barrier performance also improved significantly, with the WVP decreasing by 72.5% to 0.758 × 10^−10^ g·m/(m^2^·Pa·s), which was far below the literature median of 4.4 × 10^−10^ g·m/(m^2^·Pa·s) from the same Table. Attributable to the crack deflection and layer slippage toughening mechanisms induced by the mimicked nacre structure, the TS of the SXMZ film increased from 13.8 MPa (SW) to 62.9 MPa, representing a 356% increase, whereas the TS of similar films reported generally ranged from 12 to 32 MPa (Table 1). Regarding thermal stability, the thermal residue of the pure SW film at 800 °C was only 24.22%, whereas that of the SXMZ film increased to 36.4%, significantly higher than the maximum residue (30.59%) of the other films in Table 1. With regard to antibacterial performance, the SW, SX, and SXM films all promoted bacterial growth, while the introduction of ZnO NPs effectively reversed this trend. Ultimately, the SXMZ film achieved a 100% antibacterial rate against both *E. coli* and *S. aureus*. In summary, the modification strategy proposed in this paper effectively improved several key properties of the pure SW film. The UV-blocking performance of the resulting film was comparable to that of similar materials, while its WVP, TS, thermal stability, and antibacterial properties were superior to those of previously reported films of the same type.

### 3.11. Safety Assessment

**Figure 7:**
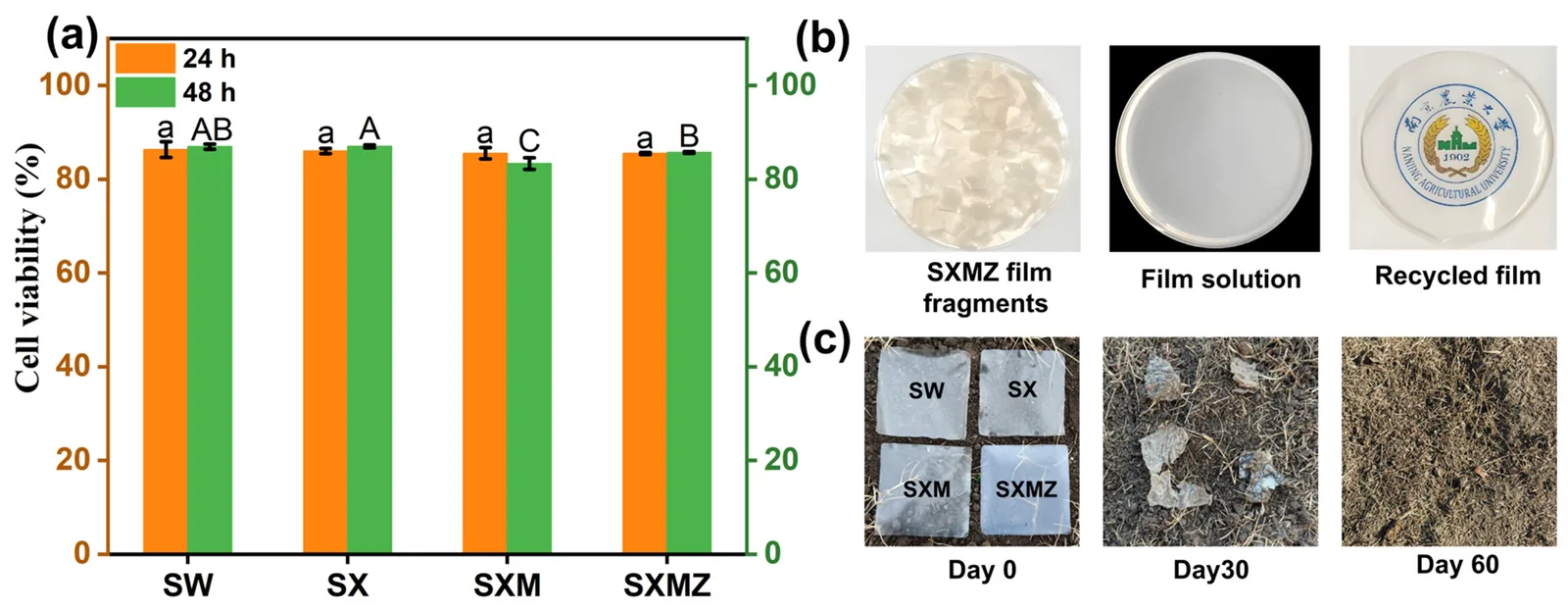
(**a**) Safety assessment, (**b**) recyclability of the SXMZ film and (**c**) soil biodegradation of the prepared film samples. For the same time point, different letters indicate significant differences between groups (*p* < 0.05). Lowercase letters and uppercase letters are used to mark 24 h and 48 h, respectively.

The safety of food packaging can be effectively evaluated through biocompatibility assessment. As depicted in Figure 7a, after co-incubation for 24 h or 48 h, all cell survival rates after treatment with films were higher than 80%, revealing that the prepared films presented no significant cytotoxicity [66]. This aligned with the findings of previous toxicity experiments on ZnO NPs composite films [67]. Another safety concern is related to the migration level of Zn^2+^. The European Plastics Regulation (EU) 2016/1416 established a specific migration limit of 5 mg of zinc per kilogram of food [68]. Previous studies reported that the migration levels of Zn^2+^ from various food packaging materials fell below this regulatory limit and were generally considered to pose a limited risk to human health [13,69].

### 3.12. Recyclability and Biodegradability

The recycling test was designed to evaluate the recyclability of the biomimetic film system developed in this study. The SXMZ film was exclusively selected, as it comprised all components, thereby fully demonstrating the modification strategy proposed herein. Furthermore, as the sample with the best overall performance, it was the most suitable for practical applications. Figure 7b illustrated the entire process for manufacturing recycled film, in which fragments of the discarded SXMZ film were dissolved, reformulated into a film-forming solution, and then dried again. After drying, dense recycled film with a mass of 0.498 ± 0.007 g and a thickness of 0.053 ± 0.003 mm was obtained, indicating that this system possessed satisfactory recyclability. Film recycling improved material utilization efficiency, effectively reduced costs, and mitigated environmental impact [70]. Furthermore, this strategy enabled reversible conversion between 3D printing and cast film processes, demonstrating exceptional cross-process adaptability. Table 2 lists the key performance parameters of the recycled film. It should be noted that its performance was slightly inferior to that of the original SXMZ film. The reason was that the original intermolecular forces were disrupted during the redissolution process, which was difficult to fully restore during the secondary film formation process [71]. Simultaneously, compared to the structural orientation formed during the 3D printing process, this solution-casting method lacked orientation-inducing effects, resulting in a random arrangement of mica, which reduced structural order. Furthermore, the diluting effect caused by the water added during the redissolution step of the recycling process also weakened intermolecular interactions, further affecting the film’s performance.

The degradation characteristics of the films were shown in Figure 7c. By day 30, all films had shown significant signs of fragmentation but had not completely degraded. However, by day 60, no continuous film structure was observed in any of the samples, indicating that the systems could degrade effectively in a soil environment. This phenomenon stemmed from the systems’ natural polysaccharide base, which gradually decomposed and was absorbed by the environment under the influence of moisture and microorganisms. The biomimetic “nacre” structure provided the necessary stability and functional properties during use but did not alter the material’s ultimate biodegradability.

### 3.13. Preservation Validation of Strawberries

**Figure 8:**
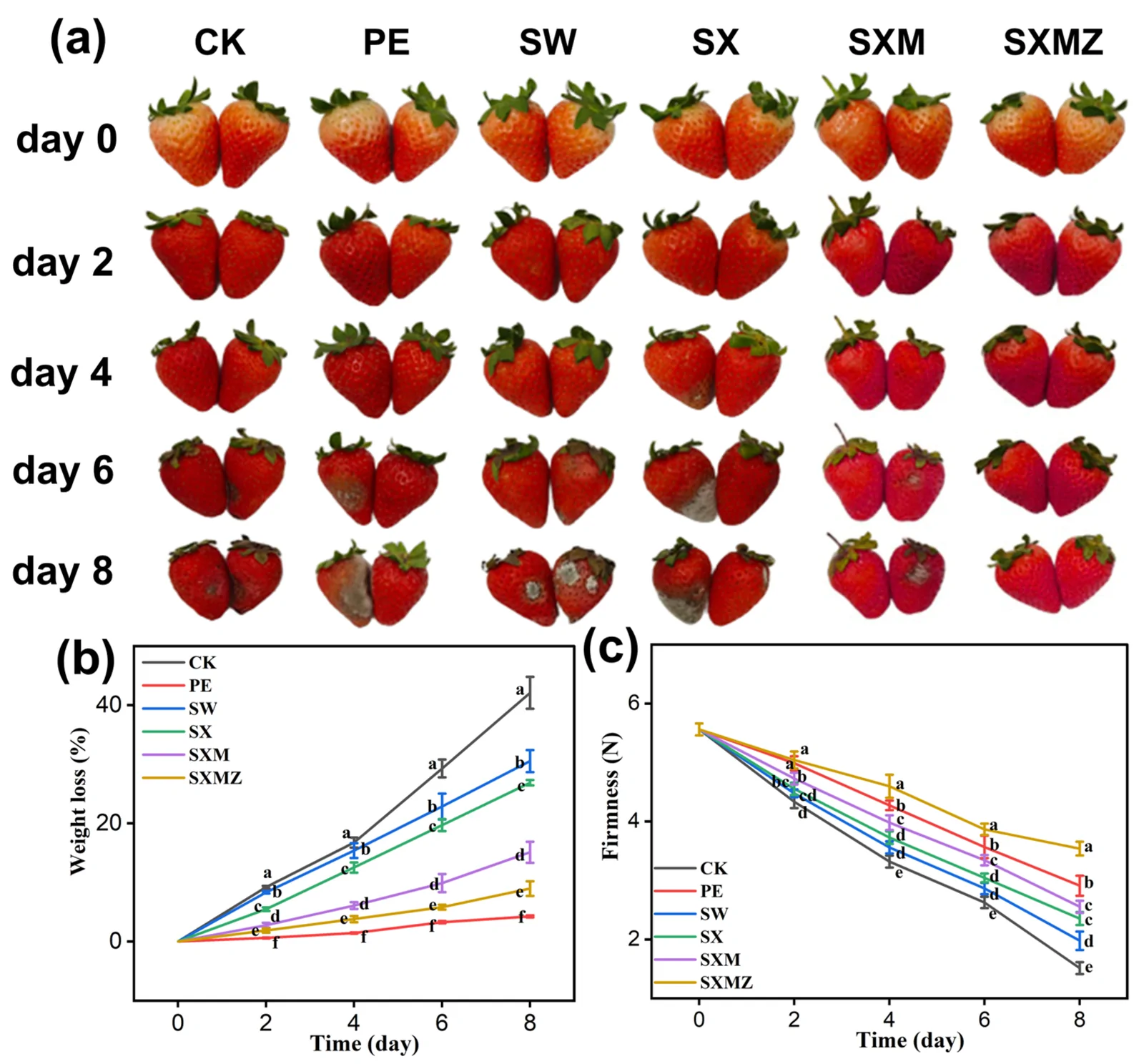
(**a**) Variations in appearance, (**b**) mass loss, and (**c**) firmness of strawberries stored under different film treatments. Different letters indicate significant differences (*p* < 0.05) among the film treatment groups at the same time point.

This study used strawberries as the test fruit and evaluated the films’ freshness-preserving performance based on appearance, weight loss, and firmness. This study used strawberries as the test fruit and evaluated the films’ freshness-preserving performance based on appearance, weight loss, and firmness. As presented in Figure 8a, the appearance of strawberries deteriorated rapidly during storage. On day 8, the fruits in the CK group and other treated groups had become noticeably shriveled and showed signs of mold growth, whereas the fruits in the SXMZ-treated group retained a relatively intact shape, exhibited the lowest degree of decay, and demonstrated significantly better overall quality. Consistent with the variations in appearance, the CK group’s weight loss (Figure 8b) had risen to 42.09% by day 8, whereas that of the SXMZ group was only 8.96%, indicating that this composite film significantly inhibited water loss. Although treatment with polyethylene (PE) film further reduced the weight loss rate to 4.25%, Figure 8c shows that fruit firmness remained at only 2.91 N, which was lower than the 3.54 N observed in the SXMZ group, indicating that PE film offered relatively limited tissue protection. Overall, this may be related to the lack of antimicrobial activity in PE film, and its excessive barrier properties limit gas exchange, resulting in an unfavorable storage gas environment [72]. In contrast, the moderate gas permeability and antibacterial activity of the SXMZ film were more effective at regulating the fruit’s microenvironment, thereby reducing water loss and delaying rot. This antibacterial effect was primarily attributed to the non-contact antibacterial mechanism of ZnO NPs [73], whereby ROS and Zn^2+^ released from ZnO NPs exerted a bactericidal effect. The absence of noticeable mold growth on the strawberries in the SXMZ group shown in Figure 8a further corroborates this result.

## 4. Conclusions

Inspired by the structure of nacre, we employed additive manufacturing technology to control the orientation of mica flakes via shear forces and fill the interlayer pores with ZnO NPs, thereby producing a dense layered sodium alginate-based composite film (SXMZ). During the construction of the nacre-like structure, hydrogen bonds were formed between the components, reducing the exposure of hydrophilic groups and thus effectively lowering the WS of the film. In addition, the nacre-like structure enhanced mechanical properties through crack deflection and layer sliding mechanisms, while also creating a tortuous permeation pathway to improve barrier performance. The introduction of ZnO NPs into the system further endowed the film with antibacterial properties. Benefiting from the above mechanisms, the film exhibited superior WVP, TS, thermal stability, and antibacterial performance compared to similar films. Moreover, the film also possessed excellent biodegradability, recyclability, and biosafety, and when applied in practice, it extended the shelf life of strawberries to 8 days. In summary, the strategy of combining biomimetic structures with 3D printing overcame the shortcomings of the SW film’s poor water resistance, barrier properties, low strength, and limited functionality. This provided a feasible design approach for degradable bio-based composite films to replace petroleum-based packaging materials.

## Figures and Tables

**Table 1:** Comparison of SXMZ film’s functions with other similar studies.

Samples	UV Barrier (280 nm, %)	WVP (10^−10^ g·m/m^2^·Pa·s)	TS (MPa)	Residual Mass (%)	Antibacterial Activity (%)	References
SXMZ	99.37	0.758	62.9	41.9 (600 °C) 36.4 (800 °C)	100 (*E. coli*) 100 *(S. aureus*)	This work
XG_25_/ZnO_10_	96	9.14	32.43	25 (800 °C)	85.90 (*E. coli*) 88.16 (*S. aureus*)	[56]
CXG-1.5	99.6	2028	2.69	28 (600 °C)	/	[44]
CTS-ZnO NPs-TTOL	/	/	42.26	30 (800 °C)	91.33 (*E. coli*) 90.67 (*S. aureus*)	[57]
5%CS@ZnONPs/LEC-Mt	100	/	9	25.95 (600 °C)	97 (*E. coli*) 97 (*S. aureus*)	[58]
FSG/SA/ CV@ZIF-8	78.7	1.18	16.75	25.06 (800 °C)	78.4 (*E. coli*) 84.42 (*S. aureus*)	[25]
SSPS/TTEO /nZnO	100	3.33	3.9	27 (600 °C)	/	[59]
GG-MN/KG-CA-BPFA	/	1.2	12.3	30.59 (800 °C)	/	[60]
SPI-GS3	/	4	13.76	22 (600 °C)	/	[61]
SPI-MA5-F	/	4.8	13.77	/	/	[62]
PS/PVA/COPE10%	/	/	5.93	/	13.5 (*E. coli*) 24 (*S. aureus*)	[63]
GA-BA	100	207.48	16	/	/	[64]
Gelatin/ZnO/CEO	100	/	32.7	12.73 (500 °C)	/	[65]

Note: Some of the values in this table are derived from figures in the cited references and may differ slightly from the original data. Readers are encouraged to consult the original literature for precise raw data.

**Table 2:** Key performance parameters of recycled film.

WS (%)	WCA (°)	UV Blocking Rate at 280 nm (%)	WVP (10^−11^ g·m/m^2^·Pa·s)	TS (MPa)	EAB (%)
68.03 ± 1.46	62.55 ± 1.42	87.87 ± 0.68	20.43 ± 1.02	19.98 ± 1.75	15.03 ± 1.19

## Data Availability

The original contributions presented in this study are included in the article material. Further inquiries can be directed to the corresponding author.

## Abbreviations

The undefined abbreviations in Table 1 are listed below:

/	Corresponding data were not provided in the original literature, or direct comparison was not possible due to differences in measurement methods
XG_25_/ZnO_10_	A composite film prepared using ultrasonically modified xanthan gum as the matrix and incorporating 10% (by weight of xanthan gum) ZnO NPs
CXG-1.5	A chitosan-xanthan gum composite film incorporating 1.5% corn mint essential oil (*v*/*v*)
CTS-ZnONPs-TTOL	A composite film composed of chitosan, ZnO NPs and tea tree oil liposomes
5%CS@ZnONPs/LEC-Mt	A composite film composed of chitosan, 5% ZnO NPs and lecithin-modified montmorillonite
FSG/SA/CV@ZIF-8	A composite film based on fish scale gelatin and sodium alginate matrix incorporated with carvacrol-loaded ZIF-8 nanoparticles (ZIF-8 is a metal–organic framework coordinated by Zn^2+^ and 2-methylimidazole)
SSPS/TTEO/nZnO	A composite film consisting of soluble soybean polysaccharide, tea tree essential oil and ZnO NPs
GG-MN/KG-CA-BPFA	A composite film consisting of agar, mica nanosheets, konjac glucomannan, carrageenan, and mulberry anthocyanins
SPI-GS3	A composite film constituted by soy protein isolate and grape seed extract
SPI-MA5-F	A composite film based on soy protein isolate and green microalgae
PS/PVA/COPE10%	A composite film formed with potato starch, polyvinyl alcohol and curcumin-oregano essential oil Pickering emulsion
GA-BA	An indicator label made from chitosan, ovalbumin, gallic acid, and blueberry anthocyanins
Gelatin/ZnO/CEO	A composite film assembled from bovine gelatin, zinc oxide and clove essential oil
